# Integrating Confidence, Difficulty, and Language Model Calibration for Better Explainability in Clinical Documents Coding: Applications of AI

**DOI:** 10.2196/78764

**Published:** 2026-04-22

**Authors:** Mihai Horia Popescu, Kevin Roitero, Vincenzo Della Mea

**Affiliations:** 1 Department of Mathematics, Computer Science and Physics (DMIF), University of Udine, via Delle Scienze, 206, Udine, 33100, Italy, 39 0432 558400

**Keywords:** deep learning, cause of death prediction, model confidence, instance difficulty, semantic, machine learning, saliency maps, prediction

## Abstract

**Background:**

In recent years, there has been increasing interest in developing machine and deep learning models capable of annotating clinical documents with semantically relevant labels. However, the complex nature of these models often leads to significant challenges regarding interpretability and transparency.

**Objective:**

This study aims to improve the interpretability of transformer models and evaluate the explainability of a deep learning–based annotation of coded clinical documents derived from death certificates. Specifically, the focus is on interpreting and explaining model behavior and predictions by leveraging calibrated confidence, saliency maps, and measures of instance difficulty applied to textualized representations coded using the International Statistical Classification of Diseases and Related Health Problems (ICD). In particular, the instance difficulty approach has previously proven effective in interpreting image-based models.

**Methods:**

We used disease language bidirectional encoder representations from transformers, a domain-specific bidirectional encoder representations from transformers model pretrained on ICD classification-related data, to analyze reverse-coded representations of death certificates from the US National Center for Health Statistics, covering the years 2014 to 2017 and comprising 12,919,268 records. The model inputs consist of textualized representations of ICD-coded fields derived from death certificates, obtained by mapping codes to the corresponding ICD concept titles. For this study, we extracted a subset of 400,000 certificates for training, 100,000 for testing, and 10,000 for validation. We assessed the model’s calibration and applied a temperature scaling post-hoc calibration method to improve the reliability of its confidence scores. Additionally, we introduced mechanisms to rank instances by difficulty using Variance of Gradients scores, which also facilitate the detection of out-of-distribution cases. Saliency maps were also used to enhance interpretability by highlighting which tokens in the input text most influenced the model’s predictions.

**Results:**

Experimental results on a pre–fine-tuned model for predicting the underlying cause of death from reverse-coded death certificate representations, which already achieves high accuracy (0.990), show good out-of-the-box calibration with respect to expected calibration error (1.40), though less so for maximum calibration error (30.91). Temperature scaling further reduces expected calibration error (1.13) while significantly increasing maximum calibration error (42.17). We report detailed Variance of Gradients analyses at the ICD category and chapter levels, including distributions of target and input categories, and provide word-level attributions using Integrated Gradients for both correctly classified and failure cases.

**Conclusions:**

This study demonstrates that enhancing interpretability and explainability in deep learning models can improve their practical utility in clinical document annotation. By addressing reliability and transparency, the proposed approaches support more informed and trustworthy application of machine learning in mission-critical medical settings. The results also highlight the ongoing need to address data limitations and ensure robust performance, especially for rare or complex cases.

## Introduction

### Background

Over the past decade, machine learning (ML) models have been increasingly gaining trust from stakeholders due to their better and better performance. This trend is even more remarkable due to the recent advances in deep learning, which have dramatically improved neural network (NN) accuracy, gaining interest over traditional techniques. As a result, NNs have seen wide adoption in a range of applications, such as health care, object detection, speech recognition, and finance; however, they are still frequently criticized for being black boxes. To gain trust from both researchers and end users in these settings, it is beneficial to develop interpretable models. Sensitive domains and real-world decision-making systems require not only accuracy on familiar data distributions but also mechanisms to ensure reliability, highlight potential errors [1], and identify uncertainty in out-of-distribution (OOD) scenarios [2]. For example, in digital health care, the system should recognize and specify when the model suggestion is poorly confident, so the control can pass to a human doctor [3,4], since for clinical scenarios, one wishes to avoid failure at all costs. Alternatively, vision models are increasingly used in safety-critical applications such as autonomous driving [5], where the detection network needs to predict the presence or absence of immediate obstructions, and the car, based on the confidence of the prediction, should decide if it should rely more on the output of other sensors or the current prediction for braking. As a result, a network should provide calibrated confidence where the probability associated with the prediction should reflect its ground truth correctness likelihood. At the same time, calibration alone does not guarantee overall reliability, as a model may be well-calibrated, yet still inaccurate or biased. Nonetheless, achieving good calibration remains an essential property of trustworthy systems, as it enables downstream decision modules to interpret predictions in a consistent and meaningful way.

Calibrated confidence is a desired feature of NNs, since good confidence estimates can be used to accomplish model interpretability. Good probability estimates provide beneficial extra information to establish trustworthiness with the user [3]; therefore, humans have a natural cognitive intuition for probabilities [6]. In addition, NNs can be incorporated into other probabilistic models by using good probability estimates [7,8]. Calibrated models have also been shown to be useful for detecting OOD data [9,10] and to improve model fairness [11].

While the main focus of these studies has been on improving the predictive accuracy of models, not much has been done on the interpretability of models, with less attention on its calibration. Initially, the models were not as accurate as today, but generally, NNs were producing well-calibrated probabilities on binary classification tasks [12]. A few years ago, many instances of miscalibration in modern NNs were reported [1], with a trend that suggests newer, larger, and more accurate models may produce poorly calibrated predictions. Recently, Minderer et al [4] revisited this question with the recent state-of-the-art image classification models and suggested that recent models such as the nonconvolutional multilayer perceptrons mixer [13] and vision transformers [14] are among the best calibrated, notably those not using convolution.

Presenting a subset of data points that a model considers to be relatively more challenging to learn can help in reasoning about model behavior. For this task, a ranking mechanism can help achieve interpretability by ranking instances by their difficulty. A mechanism for such a purpose is the Variance of Gradients (VoG) [15], which ranks data by difficulty and helps to identify the most challenging subset of instances.

Ranking mechanisms are valuable for identifying difficult instances and are also applied in detection domain analyses, including anomaly detection, OOD detection, and adversarial detection [16-19]. In particular, on classification tasks, OOD detection is very important, since it determines whether an input is in-distribution (ID) or OOD. It is unrealistic to assume that all inputs encountered in real-world settings are ID; therefore, training a model that performs perfectly on all possible inputs is inherently challenging, particularly given that real-world datasets are limited in scope. However, OOD uncertainty estimation is very challenging on modern deep learning algorithms, since it can easily produce overconfident predictions also on OOD inputs [2]. This phenomenon makes the distinction between ID and OOD data even more challenging when the dataset is not well-balanced. While there are different techniques to handle OOD detection, some authors focus on deriving OOD uncertainty measurements from the activation space of the NN by using model output [20,21] or feature representations [22]. Others instead make use of the gradient space [2]; in particular, VoG was shown to be a reliable score as an OOD detection technique [15].

Saliency maps based on gradients represent a powerful tool for enhancing text interpretability in NNs. They fall under the broader category of attribution algorithms, which aim to understand how various features, neurons, and layers contribute to a model’s output. Saliency maps specifically focus on highlighting the parts of the input data—such as tokens in transformer-based techniques—that have the most significant impact on the model’s decision. In practice, saliency maps are generated by computing the gradient of the model’s output with respect to the input text. This process reveals how small changes in the input text would affect the output. Consequently, words or phrases that cause significant changes are considered more important for the model’s predictions. This method aligns with human cognitive processes, as it provides a direct way to visualize which parts of the text are influencing the model’s decisions [23,24].

In this paper, we experiment with the proposed techniques in a medical classification task, namely, the identification of the so-called underlying cause of death (UCOD) from death certificates. The UCOD was defined by the World Health Organization (WHO) as “I (a) the disease or injury which initiated the train of morbid events leading directly to death; or (b) the circumstances of the accident or violence which produced the fatal injury,” together with the rules for its selection [25]. In principle, this important condition is part of the stated information in the death certificate; yet, it is not always easy to identify. As of today, few rule-based systems are used for UCOD selection. ML has been shown to be very effective; however, such models are often designed as black boxes. Therefore, without explainability of the results, the experts are not willing to adopt such models.

In this work, we build upon a previously developed deep learning model that achieved state-of-the-art performance in UCOD identification [26]. While the model demonstrates strong predictive accuracy, its reliability and interpretability have not yet been systematically examined, particularly with respect to calibration and the explainability of its predictions.

### Related Work

#### Model Calibration

While ML models are not always well calibrated, many techniques have been proposed to train NNs for calibration or post-hoc calibrate a model without losing the focus of improving the predictive accuracy. According to Karandikar et al [27], the existing calibration approaches can be categorized into the following 3 categories. The first category explicitly rewards calibration by augmenting or replacing the primary training loss such as accuracy versus uncertainty calibration loss [28], maximum mean calibration error loss [29], and focal loss [30]. Recently, Hui and Belkin [31] have shown that using the mean square error loss would further improve the performance, and no extra loss rescaling parameters would be necessary, as was required for the cross-entropy loss. While Karandikar et al [27] suggest that applying across multiple primary losses, the methods outperform all these calibration-incentivizing training objectives.

A second category of methods examines model changes, such as deep ensembles, for predictive uncertainty estimation. In Lakshminarayanan et al [20], it is shown that this approach is a strong baseline on evaluation metrics and is a simple and effective method for ensembling. This approach trains multiple copies of a network and aggregates the individual models to form a mixture distribution on which the predictions are made. Wen et al [32] proposed batch ensemble, a computationally efficient ensembling method that maintains competitive accuracy and uncertainty estimates while significantly reducing training and inference costs and scaling effectively to lifelong learning scenarios. Recent work of Dusenberry et al [33] has shown a prior distribution as a simple strategy for aggregating multimodal weight solutions, similar to deep ensembles.

A third category consists of post-hoc calibrating a model by rescaling the model predictions after training. The most popular technique for this category is temperature scaling [1], which maximizes a single temperature parameter on held-out negative log likelihood. Regarding calibration of pretrained transformers in natural language processing, Desai and Durrett [34] analyzed the calibration on 2 models, bidirectional encoder representations from transformers (BERT) [35] and robustly optimized bidirectional encoder representations from transformers approach (ROBERTa) [36], across 3 tasks. When used out of the box, the authors show that the pretrained models are well calibrated in-domain, but out-of-domain calibration can be as much as 3.5 times lower. The authors suggest that temperature scaling post-hoc calibration can further reduce calibration error in-domain, and using label smoothing helps calibrate posteriors out-of-domain. Similar to previous work [34], we focus on post-hoc calibration of transformers by studying the performance of an in-domain model trained by the method disease language bidirectional encoder representations from transformers (DiLBERT) [37], checking improvements by using temperature scaling.

In addressing model calibration, we specifically opt for post-hoc calibration methods, since we prioritize the preservation of the original training procedure and model integrity. This approach has been chosen to focus on transparent and nonintrusive adjustments, thus avoiding direct optimization methods that could potentially compromise model effectiveness on the task.

It is important to note that our methodology is reliant on the use of encoder-based models. This is due to the inherent compatibility of encoder-based models with the state-of-the-art calibration techniques, which rely on logit confidence scores. Consequently, our study does not incorporate models like LlamA [38] or Mistral [39], as these models are causal autoregressive in nature, which does not align with our requirement for accessible and adjustable logit confidence scores. Additionally, we do not use ChatGPT as part of the experimental or modeling pipeline because of its proprietary nature and the challenges associated with conducting transparent and replicable research with such closed systems.

#### Estimating Instance Difficulty

Presenting a subset of data points that a model considers to be relatively more challenging to learn can help in reasoning about model behavior. Techniques aimed at identifying such behavior can be categorized based on their objectives. Case-based reasoning not only aids interpretability [40,41] but also facilitates the identification of atypical examples for human auditing [42] and enables the model to refrain from classifying uncertain instances [43,44]. Human auditing can be challenging for large datasets; therefore, prior work has focused on methods that automatically identify a subset of more challenging examples to prioritize limited human annotation and auditing resources. For such a task, an effective tool is saliency maps [45]; additionally, several recent studies have focused on the problem of estimating example difficulty by using a ranking mechanism such as VoG [15]. From their study, the authors suggest that the images that appear more difficult also have a higher VoG score. For each image, the algorithm is calculating the gradient of the activations with respect to the pixels over multiple checkpoints during the training process. The VoG score is calculated as the average (over pixels) of the per-pixel variance across these checkpoints. Using gradient information, Kokilepersaud et al [46] have proposed Gradient Constraint, where they have been using gradient measures as a method to detect anomalies by assigning pseudoseverity labels to a large set of unlabeled optical coherence tomography scans.

Another approach to estimating example difficulty relies on prediction depth [47], which provides an alternative perspective compared with gradient-based methods. The prediction depth represents the first hidden layers, after which the k-nearest neighbor classifier can effectively classify an example using the representation of the image in all subsequent layers. The authors show that the prediction depth is larger for examples that visually appear to be more difficult. Their investigations also reveal that the predictions are on average more accurate for validation points with small prediction depths.

In addition to methodological differences, we can categorize the approaches into 2 groups based on when they score training instances: those that use the final trained model and those that assess instances early in training. Specifically, the methods developed by Agarwal et al [15] and Baldock et al [47] fall into the first category, as they use the final trained network. In contrast, the methods by Paul et al [48] belong to the second category, as they highlight the presence of a strong signal for estimating example difficulty very early in the training process. To this end, they propose scoring the importance of each training example based on its expected loss gradient norm (Gradient Normed score). Furthermore, the authors suggest that, within the first few epochs of training, the Gradient Normed score can be effectively approximated by the norm of the error vector (Error L2-Norm score). They conclude by demonstrating that significant fractions of the training data can be pruned without sacrificing test accuracy, as evidenced by their experiments across a variety of architectures and datasets.

#### Gradients Techniques Used to Identify OOD Samples

Gradients can be used alternatively for more specific tasks, such as detecting adversarial, anomalous, and OOD samples. Igoe et al [49] and Kwon et al [50] proposed the use of back-propagated gradients from NNs to obtain the model-based characterization of abnormality. The authors afterward refine the previous idea, proposing an anomaly detection algorithm using the Gradient Constraint [16]. In their experiment, the authors measure the cosine similarity between past normal gradients and the current input. Shifting focus to research themes associated with anomaly detection, we find its applications in OOD. Unlike OOD detection, which presupposes multiclass data with identifiable labels, in anomaly detection, we can distinguish between monolithic sets of normal and anomalous data. Considering OOD detection [51], make use of the Mahalanobis distance of the gradient to detect OOD samples. Instead, Lee et al [17] propose to use back-propagated gradients to characterize anomalies in inputs seen during inference from the perspective of the model. In Grad Norm [2], the authors propose a gradient-based OOD uncertainty estimation method, which is label-agnostic and where no outlier data are required. For the adversarial detection problem, in raw gradient anomaly detection, the authors analyze the temporal distribution of the entire raw gradient by their end-to-end deep learning–based architecture. In detecting adversarial attacks by analyzing gradients, Schulze et al [18] analyze the raw gradient of the last two layers of classifiers.

#### Saliency Maps

In Malkiel et al [24], a novel unsupervised method is presented to explain paragraph similarities using pretrained BERT models. The technique identifies and matches keywords across paragraph pairs, highlighting the crucial pairs that best explain their similarity. To evaluate the reliability of saliency maps for text analysis, Kokhlikyan et al [52] compare their application with image-based models. They discover that input multipliers maintain text structural patterns across different models, leading to uniform explanations. The study also highlights that smoother NN components, such as SoftPlus instead of rectified linear units, significantly enhance the accuracy and reliability of saliency-based interpretations in text. Borgnia et al [53] propose a novel saliency approach by analyzing network parameters instead of inputs to understand NN errors. The findings reveal that problematic parameters cause semantically similar misclassifications, and pruning or fine-tuning these parameters can rectify similar errors across different samples. Additionally, the authors developed a technique for linking image features to parameter malfunctions, enhancing model interpretability. The effectiveness of this method is confirmed through extensive validation, offering a fresh perspective on NN diagnostics.

Captum Insights is an open-source interpretability library designed for PyTorch [23], encapsulating a variety of gradient and perturbation-based attribution methods. This tool, suitable for a range of models beyond mere classification tasks, supports multiple data types, including images, text, and more, ensuring wide applicability. Highlighting its multimodal and extendable nature, the library aims to simplify and enhance the interpretability process. The latter is part of the interpretability techniques applied in our study.

#### UCOD Selection

The UCOD is the most important condition used for statistical comparison and public health data. To be able to correctly identify the UCOD in a death certificate, the certificate needs to undergo 2 different processes: first, the textual conditions need to be coded according to a standard defined by the WHO, using the International Statistical Classification of Diseases and Related Health Problems (ICD). The second process is the selection of the UCOD, according to specific rules defined by WHO [25]. The selection of the UCOD is a laborious process, and there is a need for automatic or semiautomatic systems to limit errors. Therefore, there is much interest in developing support systems for such a purpose. We can identify 2 methodologies for such systems: rule-based systems and ML systems. Currently, there are 3 published rule-based systems that implement the WHO instructions to select the UCOD; other proprietary and unpublished systems might exist. The most used system is Iris [54], which currently supports ICD 10th revision (ICD-10) and is being updated to support ICD 11th revision (ICD-11). Automated Classification of Medical Entities [55], the oldest one, is a system developed in the United States, which can also identify the UCOD for ICD-10. The last entrant is Digital Open Rule Integrated Cause of Death Selection [56], which is a new rule-based system designed and made available by WHO to identify the UCOD for ICD-11. Significant past work was devoted to the application of ML techniques for this specific task, reaching the state of the art over the traditional rule-based systems [26,57]. However, despite the accuracy achievements, such systems are still not officially acknowledged by mortality coding institutions and are not yet used in practice. This is due to the necessarily conservative approach that drives the collection of data for statistical purposes. In particular, using deep learning techniques, Falissard et al [57] developed a modified Inception network, obtaining an accuracy score of 0.978 on a French death certificate dataset. Pita Ferreira et al [58] conducted a sensitivity analysis of AUTOCOD, a deep learning model used to classify the UCOD from free-text certificates in Portugal, reporting an overall *F*_1_-score of 0.88 at the category level and 0.94 at the block level. In our own previous work [26], we have shown that by fine-tuning a transformer model, we can reach the state of the art by obtaining an accuracy score of 0.990. By comparison, reported Iris accuracy is 0.74‐0.78 from evaluations in the Netherlands [59] and France [57]. Falissard et al [57] also reported a score of 0.92 when considering only nonrejected certificates. Similar results were obtained in the preliminary validation of Digital Open Rule Integrated Cause of Death Selection, with an accuracy of 0.78 for ICD-10, while it achieved an accuracy of 0.63 on ICD-11 [56]. Our analysis in this paper centers on the model proposed by Della Mea et al [26].

### Objectives

This study aimed to evaluate the interpretability and reliability of a deep learning model for UCOD identification. Specifically, we assess whether the model is well calibrated and whether its calibration can be improved using temperature scaling post-hoc calibration. In addition, we investigate a mechanism to rank instances based on difficulty using VoG [15] and explore its applicability for OOD detection in textual representations.

## Methods

### Definitions

This paper addresses 2 main problems that improve the interpretability of a transformer model. The first study aims to evaluate and improve the error calibration of the model by using post-hoc temperature scaling calibration, while the second study focuses on implementing a ranking mechanism that uses VoG to estimate the difficulty of predicting instances.

#### Disease Language Bidirectional Encoder Representations From Transformers

The language model used in this study is DiLBERT [37], which is a domain-specific BERT model that has been pretrained on English disease-related corpora containing ICD-11 classification entities, Wikipedia and PubMed documents. The DiLBERT model’s architecture mirrors that of the original BERT base model. It features 12 BERT encoders stacked together, each with 12 attention heads. The hidden size of the model (and the embedding dimension) is 768. Additionally, it can handle input sequences up to 512 tokens in length. The pretraining was performed using a masked language modeling task for 50 epochs on a 3.3 billion words corpus, with a batch size of 256.

#### Posterior Calibration

A model is said to be well calibrated when the estimated confidence of the predictions is aligned with empirical likelihoods. For example, given 100 predictions, each receives a posterior probability of 0.9, it is expected that 90 predictions will be correctly classified. Formally, we consider a multiclass classification problem in which an input x ∈ X is observed, and a categorical output y∈Y={1,2,…,k} is predicted, which are random variables that follow a ground truth joint distribution π(x,y)=π(y| x)π(x). Let *f* be an NN model, the predictor *f* is modeled as a function that maps every x∈X to a categorical distribution over *k* labels, where $f\left(x\right)=\left(\hat{y},\hat{p}\right)$ is a class prediction, and $\hat{p}$ is the associated confidence, that is, the probability of correctness. Since the objective is to obtain a confidence estimate $\hat{p}$ to be calibrated, then intuitively $\hat{p}$ should represent a true probability. Perfect calibration can be defined as


(1) $$P\left(\hat{y}=y|\hat{p}=p\right)=p,\;\forall \;p\in [0,1]$$

where the probability is over the joint distribution. While 1 represents the ideal settings, in practice, achieving perfect calibration is impossible, since $\hat{P}$ is a continuous random variable, and the probability in the equation cannot be computed using finitely many samples. This motivates the need for empirical approximations; following previous work [1], one can group predictions into *M* interval bins, where the bins are equally sized (each of size 1/*M*), then calculate the accuracy per bin. Let *B_m_* be the set of samples whose prediction confidence belongs to the interval $I_{m}=\left(\frac{m-1}{M},\frac{m}{M}\right]$ [1].

#### Expected Calibration Error

Previous work in measuring calibration suggests that a common metric often used in practice is expected calibration error (ECE) [60], which is a convenient scalar summary statistic of calibration. ECE makes use of equally spaced bins and is computed as the weighted average of the difference between each bin’s accuracy and confidence. Formally, the accuracy of *B_m_* can be defined as


(2) $$acc(B_{m})=\frac{1}{\left|B_{m}\right|}\sum_{i\in B_{m}}1(\hat{y_{i}}=y_{i})$$

where $\hat{y}$ is the predicted class label for the sample *i*, and *y* is the true class label for the same sample. The average confidence of *B*_*m*_ bin can be defined as


(3) $$\text{conf}\left(B_{m}\right)=\frac{1}{|B_{m}|}\sum_{i\in B_{m}}{\hat{p}}_{i}$$

where $\hat{p_{i}}$ is the confidence of the sample *i*. Then, perfect calibration can be accomplished when ∀m∈{1,…,M}, then $\text{acc}\left(B_{m}\right)=\text{conf}\left(B_{m}\right)$ (ie, when accuracy and confidence of the model align perfectly across all intervals considered). Based on the accuracy and confidence formulations, ECE can be defined as


(4) $$\text{ECE}=\sum_{m=1}^{M}\frac{\left|B_{m}\right|}{n}\left|\text{acc}\left(B_{m}\right)-\text{conf}\left(B_{m}\right)\right|,$$

where *n* is the number of samples [1].

#### Maximum Calibration Error

As reported by Guo et al [1], ECE can be considered as the primary metric for measuring calibration. However, depending on specific requirements, other variant metrics may also be used, such as each one that emphasizes different aspects of calibration. Maximum calibration error (MCE) is one of those variants that is based on minimizing the worst-case deviation between confidence and accuracy, which can measure the reliability in high-risk applications. As in the case of ECE, the computation of MCE relies on an empirical approximation with equally spaced bins. The MCE can be defined as


(5) $$\text{MCE}=\max_{m\in \left\{1,\ldots ,M\right\}}\left|\text{acc}\left(B_{m}\right)-\text{conf}\left(B_{m}\right)\right|$$

Perfect calibration can be defined as for ECE when $\forall m\in \{1,\ldots ,M\},\;\text{acc}\left(B_{m}\right)=\text{conf}\left(B_{m}\right)$. When the model is perfectly calibrated, the values of MCE and ECE are both equal to 0 [1].

#### Temperature Scaling

Temperature scaling [1] is a post-hoc calibration method that adjusts the confidence scores produced by a deep learning model without altering its predicted class labels. It involves a single scalar parameter *T* called temperature, which is used to scale the logits of a model before applying the softmax function, which converts then the produced logits into probabilities. The primary goal of temperature scaling is to improve the calibration of the model, making the predicted probabilities more reflective of the true likelihood of correctness. Let *z*(*x*) represent the vector of logits output by the NN for an input *x*. Temperature scaling modifies these logits as *z*(*x*)/*T*, where *T* > 0 is the temperature. The temperature score is typically determined on a validation set by minimizing a calibration metric, typically ECE or MCE.

#### Variance of Gradients

VoG is used to compute an instance score, which is of great interest, that is, whether a sample is difficult (ie, challenging) for a model to classify. Such information can be leveraged during model deployment, for example, by identifying difficult instances and handling them differently, such as referring them to a human assessor. In this work, we rely on the VoG score [15], originally defined for images and conveniently modified to be used in a natural language processing problem. In our adaptation, instead of computing gradient variance over image pixels, we compute it over tokens by taking the gradient of the final activation layer with respect to each token’s embedding. The variance of these token-level gradients (computed per class) is used as the stratification variable. VoG is computed by considering, for a given instance, the gradient of the final activation layer with respect to each part of the input instance at the token level. The measure is computed separately for each class. In other terms, this measures the contribution of each single instance to the final class prediction [45]. Formally,


(6) $$S=\frac{\partial A_{p}^{l}}{\partial x_{i}}$$

represents the gradient matrix of the last layer *l* for the class *p* (ie, $A_{p}^{l}$) for each part of the instance *x*_*i*_ (ie, its tokens), then VoG can be simply computed by considering the average VoG score for each instance. To handle class imbalance, we also normalize VoG by considering the average VoG of each class.

#### Saliency Maps

To compute feature attributions for the model’s predictions, we used Integrated Gradients (IG). This method attributes importance to each input component (token in our case) by accumulating the gradients of the output with respect to the inputs along a path that interpolates between a baseline input *x*^baseline^ and the actual input *x*. The method is grounded in 2 axioms, sensitivity and implementation invariance, which ensure that the resulting attributions are faithful to the behavior of the model.

Formally, for a function *F*: *R^n^*→*R* and an input *x*, the attribution for its *i*-th dimension is defined as:


(7) $${\text{Integrated Gradients}}_{i\;}\left(x\right)=\left(x_{i}-x_{i}^{\text{baseline}}\right)\int_{\alpha =0}^{1}\frac{\partial F\left(x^{\text{baseline}}+\alpha \left(x-x^{\text{baseline}}\right)\right)}{\partial x_{i}}d\alpha$$

In practice, this path integral is approximated numerically (eg, via a Riemann sum or Gauss-Legendre), providing a stable estimate of each feature’s contribution to the model’s prediction. Further details on the theoretical properties of IG can be found in the original work [61].

The IG obtained for each input token serves as the attribution scores, which we then normalize and aggregate at the word level for visualization.

### Task and Statistical Analysis

This study uses a dataset consisting of coded death certificates obtained from the US National Center for Health Statistics (NCHS), which are available for statistical and analytical research purposes. The dataset spans across the years 2014‐2017 and includes 12,919,268 records. The selected data correspond to the pre–COVID-19 pandemic period. Data from the pandemic period were deliberately excluded because the pandemic introduced temporary ICD codes and evolving UCOD selection rules, resulting in unstable and noncomparable coding practices. Restricting the analysis to the prepandemic period ensures consistency in coding rules and provides a stable basis for the evaluation.

Each record contains administrative data such as sex, age, and concatenated conditions from both Part 1 and Part 2 of the certificate, as well as the corresponding UCOD. An example of a death certificate is presented in Table 1. The original records are already provided in an ICD-10 coded format, as the US National Center for Health Statistics public database does not contain the original free-text narratives written by physicians for privacy and anonymization reasons.

**Table 1.** Example of a coded death certificate.

	Condition
Part 1	
1	I21.9 Acute myocardial infarction
2	I10 Hypertension
3	N19 Unspecified kidney failure
4	*—* ^a^
Part 2	
1	—^a^
Other	
Administrative data	Sex: female, age: 55 years
Underlying cause of death	I21.9 Acute myocardial infarction

a Not applicable.

As UCOD selection is generally a two-step process: (1) coding the free text into ICD codes and (2) applying the mortality coding rules to select the UCOD—this study focuses exclusively on the second step, as the first step cannot be studied with this dataset.

To enable the use of textual data, we performed reverse coding from the death certificate to its textual description, converting the administrative data into a textual format and mapping each code to its corresponding ICD title. The resulting encoding is illustrated with 3 examples in Table 2. For this study, we extracted a subset of 400,000 certificates for training, 100,000 for testing, and 10,000 for validation purposes from the total number of records in the dataset. This split was selected to ensure comparability with prior work using the same experimental setup, while also reflecting practical computational constraints, as the evaluation involved training and comparing multiple (7) alternative models. Extraction has been carried out with stratified sampling to have a subset matching the features of the entire dataset.

**Table 2.** Example encoding for the death certificates as a sentence.

Every record of the death certificate as a single sentence	UCOD^a^
Female, 55 years: (Acute myocardial infarction) due to (Hypertension) due to (Unspecified kidney failure)	I21.9
Male, 39 years: (Malignant neoplasm of breast, unspecified or malignant neoplasm, without specification of site)	C50.9
Male, 40 years: (Sepsis, unspecified) due to (Necrotizing fasciitis)	M72.6

a UCOD: underlying cause of death.

We have pretrained and fine-tuned DiLBERT [37] on the earlier-mentioned dataset. One aspect worth mentioning is that ICD-10 is partitioned into 22 chapters according to the disease site or etiology. In addition to the overall performance of the model, we wanted to analyze the performance by disease category, based on the ICD-10 chapter organization. Thus, the accuracy of the DiLBERT model at the chapter level is presented in Table 3, along with the frequency of the target coded condition as a chapter and the frequency of the chapter considering the input conditions coded. These statistics consider both training and test records.

The table highlights some interesting observations. For example, Chapter XXI is not relevant for mortality and does not appear in any of the death certificates. Chapter XXII, on the other hand, is reserved for special purposes, such as emergency situations, and codes are typically added to this chapter before they are assigned to their appropriate classification in subsequent years. Notably, during the COVID-19 pandemic, a special code for the disease was added to Chapter XXII.

**Table 3.** Accuracy by ICD-10^a^ chapter (table sorted according to ICD-10 chapter field), frequency of each chapter as target and input coded conditions.

ICD-10 chapter	Input frequency, n (%)	Target frequency, n (%)	Accuracy @1
I Certain infectious and parasitic diseases	12,096 (2.521)	48,384 (3.283)	0.925
II Neoplasms	106,126 (22.118)	148,271 (10.060)	0.986
III Diseases of the blood and blood-forming organs and certain disorders involving the immune mechanism	12,624 (0.857)	1,592 (0.332)	0.794
IV Endocrine, nutritional, and metabolic diseases	86,320 (5.857)	21,124 (4.402)	0.952
V Mental and behavioral disorders	116,318 (7.892)	23,239 (4.843)	0.976
VI Diseases of the nervous system	57,602 (3.908)	35,094 (7.314)	0.981
VII Diseases of the eye and adnexa	444 (0.030)	5 (0.001)	—^b^
VIII Diseases of the ear and mastoid process	131 (0.009)	6 (0.001)	0
IX Diseases of the circulatory system	458,620 (31.116)	148,518 (30.953)	0.978
X Diseases of the respiratory system	172,966 (11.735)	48,674 (10.144)	0.978
XI Diseases of the digestive system	48,211 (3.271)	18,119 (3.776)	0.941
XII Diseases of the skin and subcutaneous tissue	4,257 (0.289)	821 (0.171)	0.892
XIII Diseases of the musculoskeletal system and connective tissue	12,300 (0.835)	2,345 (0.489)	0.819
XIV Diseases of the genitourinary system	65,914 (4.472)	12,196 (2.542)	0.960
XV Pregnancy, childbirth, and the puerperium	651 (0.044)	215 (0.045)	0.581
XVI Certain conditions originating in the perinatal period	5,570 (0.378)	1,964 (0.409)	0.805
XVII Congenital malformations, deformations, and chromosomal abnormalities	3,125 (0.212)	1,644 (0.343)	0.708
XVIII Symptoms, signs, and abnormal clinical and laboratory findings, not elsewhere classified	91,947 (6.238)	6,142 (1.280)	0.985
XIX Injury, poisoning, and certain other consequences of external causes	87,478 (5.935)	—	—
XX External causes of morbidity and mortality	52,752 (3.579)	39,906 (8.317)	0.962

a ICD-10: International Statistical Classification of Diseases and Related Health Problems 10th Revision.

b Not available.

An analysis of the accuracy of the DiLBERT model at the chapter level reveals that chapters with a target frequency of less than 1% have an accuracy score below 0.9, while chapter VIII, with a frequency of less than 0.01%, has an accuracy score of 0. Chapter VII also has a frequency of less than 0.01% in the training set but has no cases in the test set. Chapter XIX, which covers injury, poisoning, and certain other consequences of external causes, is unique in that it has a relatively high input frequency of 5.9%; yet, it is never selected as the UCOD by the model. This is because such conditions are considered consequences of an external cause, and the external cause itself (ie, Chapter XX) is always the cause that should be selected as the UCOD. Therefore, the frequency of external causes as a target is higher than the input frequency, as these causes of death are more likely to be selected when present in the certificate. Similarly, for neoplasms (ie, Chapter II), the input frequency is higher than the target frequency because when a malignant neoplasm is the UCOD, multiple malignant neoplasms may be stated on the same death certificate.

While there are codes that cannot be selected as the UCOD and are scattered across different chapters, there are certain conditions that are more likely to be excluded. For example, the entire Chapter XVIII (Symptoms, signs, and abnormal clinical and laboratory findings, not elsewhere classified) is part of the so-called ill-defined conditions. These conditions are typically not selected as the UCOD if other conditions that are not ill-defined are present on the certificate, which is why Chapter XVIII has a very small target frequency compared to the associated input frequency.

While ICD-10 contains more than 14,000 codes, not all of them can be used for mortality coding and the selection of the UCOD (eg, Z codes). It is difficult to estimate the number of codes missing from our dataset since we cannot identify all the codes that can be used for mortality coding. For instance, some codes may not be present because the distribution of diseases varies between regions, and our dataset only includes records from the United States. Furthermore, physicians may not always use the most precise condition on the death certificate, resulting in the use of residuals (identified by 0.9 in ICD-10) instead of the most precise code, as shown in Tables4  5.

The dataset used in this study contains 2066 different codes stated as the target class and 3999 different codes stated as coded conditions on the death certificates. The top 10 UCODs with their frequency are presented in Table 4, covering approximately 36% of the total number of records. Interestingly, as shown in Table 5, the top 10 conditions stated on the death certificates differ from the selected UCODs in Table 4. The most commonly used conditions have a frequency of 31.2% of the total conditions stated.

**Table 4.** Top 10 most selected underlying cause of death (UCOD).

UCOD	Title	Values, n (%)
I25.1	Atherosclerotic heart disease	26,523 (5.527)
C34.9	Bronchus or lung, unspecified	26,232 (5.466)
J44.9	Chronic obstructive pulmonary disease, unspecified	21,504 (4.481)
I21.9	Acute myocardial infarction, unspecified	20,176 (4.204)
G30.9	Alzheimer disease, unspecified	19,598 (4.084)
F03	Unspecified dementia	18,135 (3.779)
I50.0	Congestive heart failure	11,882 (2.476)
I64	Stroke, not specified as hemorrhage or infarction	10,757 (2.241)
I25.0	Atherosclerotic cardiovascular disease, so described	10,406 (2.168)
J18.9	Pneumonia, organism unspecified	7,679 (1.600)

**Table 5.** Top 10 most stated conditions on the death certificate.

Condition stated	Title	Values, n (%)
I46.9	Cardiac arrest, unspecified	62,926 (4.269)
I10	Essential (primary) hypertension	62,666 (4.251)
F17.9	Mental and behavioral disorders due to use of tobacco	54,675 (3.709)
I25.1	Atherosclerotic heart disease	52,057 (3.531)
J44.9	Chronic obstructive pulmonary disease, unspecified	50,101 (3.399)
I50.0	Congestive heart failure	49,042 (3.327)
F03	Unspecified dementia	34,343 (2.330)
A41.9	Sepsis, unspecified	33,818 (2.294)
J96.9	Respiratory failure, unspecified	31,180 (2.115)
J18.9	Pneumonia, unspecified	28,525 (1.935)

### Ethical Considerations

This study did not involve human participants or any form of human subject research. The analyses were conducted exclusively on publicly available, anonymized death certificate data, which contain no personally identifiable information. No attempt was made to reidentify individuals, and no procedures, interventions, or interactions with human subjects were performed; therefore, none of the ethical review elements listed in the JMIR guidelines apply.

## Results

### Model Calibration

**Figure 1.**
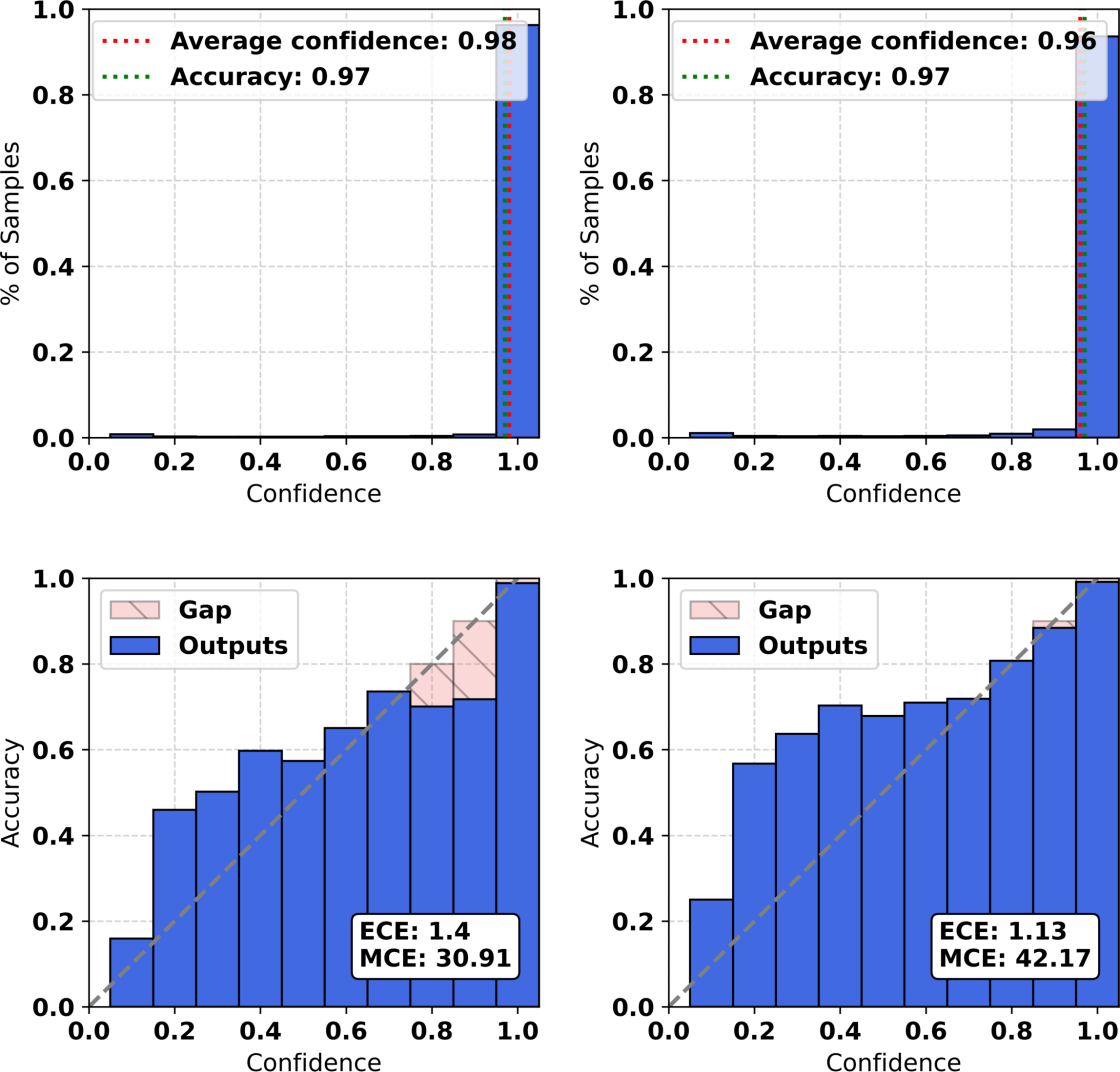
Confidence histograms (top) and reliability diagrams (bottom), for out-of-the-box (left) and with temperature scaling (right). ECE: expected calibration error; MCE: maximum calibration error.

The top row of Figure 1 shows the distribution of prediction confidence, while the bottom row shows the so-called reliability diagram, which plots accuracy as a function of confidence. The reliability diagram provides a visual representation of model calibration, with perfect calibration represented by plotting the identity function (ie, *x*=*y*, represented by the dotted line in the figure). Following Guo et al [1], confidence scores are grouped into bins, and for each bin, the average predicted confidence is compared with the corresponding empirical accuracy. The reliability diagram highlights, in red, the discrepancy between these 2 quantities, providing a visual indication of model miscalibration. Thus, deviations from the perfect diagonal indicate model miscalibration. The figure includes results for the out-of-the-box samples in the left column and samples rescaled with temperature scaling in the right column.

Overall, the histograms and reliability diagram shown in Figure 1 demonstrate that the out-of-the-box average confidence of the predictions closely matches their accuracy, with confidence slightly higher than accuracy, indicating a small miscalibration. The bottom left panel shows that most of the bins are not well calibrated, as indicated by the high MCE of 30.91. However, the ECE is low, with an error of 1.4, suggesting that the model is well calibrated overall. This is partly due to the last bin, which is both well-calibrated and represents the highest number of samples.

When temperature scaling is applied, the bins with high confidence become well calibrated, but those with low and medium confidence become less calibrated, as shown in the bottom right panel of Figure 1. The temperature scaling method reduces the ECE to 1.13, while the MCE increases to 42.17. It is important to note that, while there is an observed increase in the MCE metric, this increment is relatively small. More significantly, the key aspect of the calibration phase can be seen in the improved calibration scores for the instances in the right-most part of the plot, specifically those having a confidence level greater than or equal to 0.8. This enhanced calibration at higher confidence levels is a crucial factor in developing a reliable model, particularly for the most practically interesting instances, that is, those having high-confidence scores. As reported in Desai and Durrett [34], pretrained models are generally well calibrated, although further improvements are possible; in this case, the out-of-the-box ECE was already very low.

### Confidence Reliability

**Figure 2.**
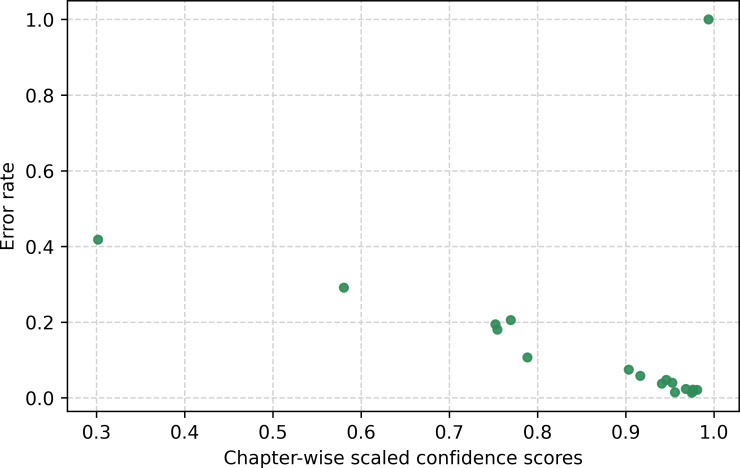
Chapter-wise scaled confidence with error rate.

**Figure 3.**
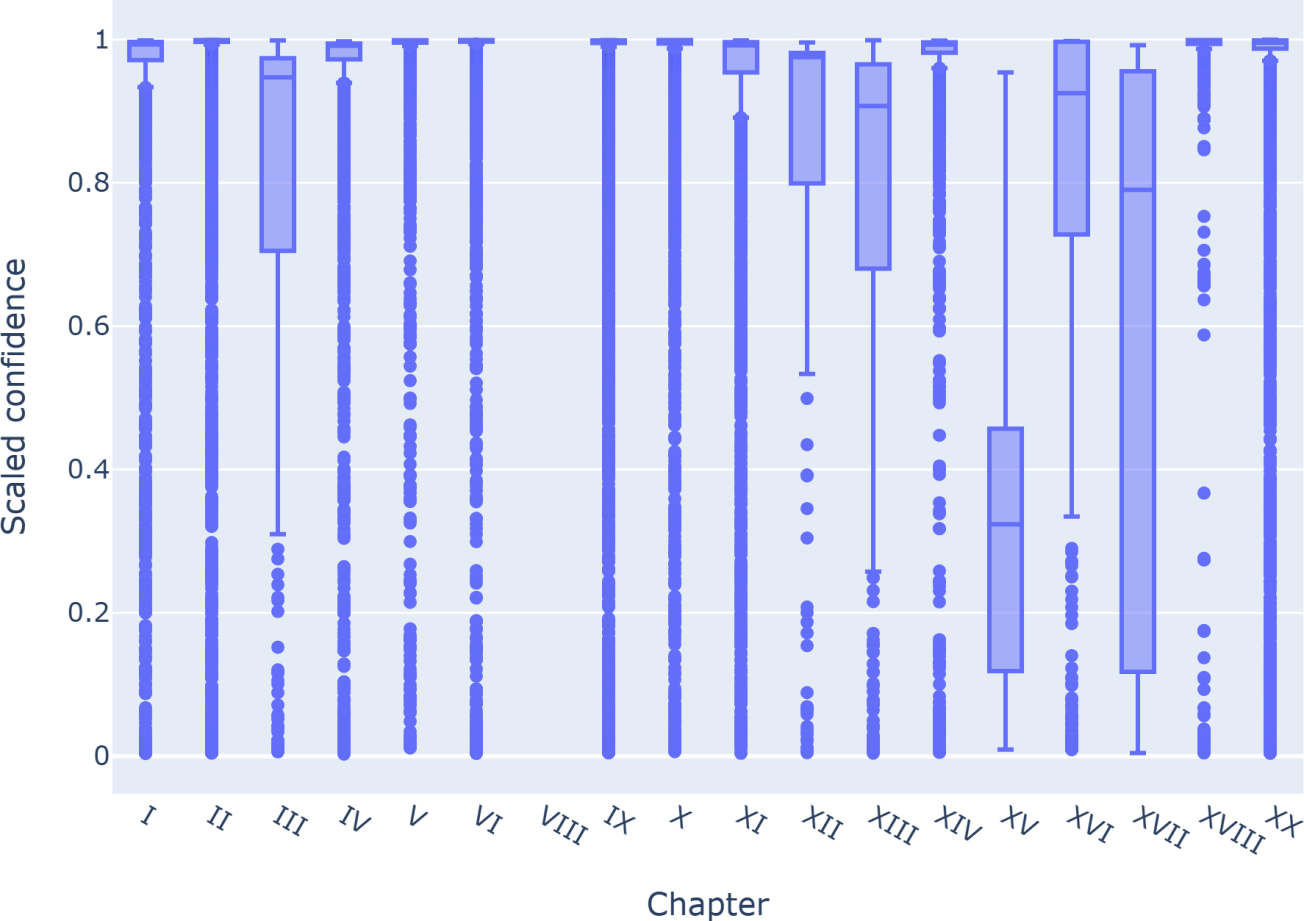
Class-wise scaled confidence percentiles per chapter.

A well-calibrated model can use its confidence as a measure of prediction reliability, which is often used to focus on chapter granularity or abstract ranges of conditions in UCOD predictions. Figure 2 shows the error rate as a function of confidence for instances, grouped by chapter. Excluding the chapter with a 100% error rate and a confidence of 1 (VIII), we observe a correlation between confidence and error rate at the chapter level. Figure 3 displays the scaled confidence distribution of the instances by chapter, with data quartiles and averages. The plot shows that half of the chapters have a very low IQR, indicating that the model is highly decisive in predicting those chapters, with quartile groups close to a confidence of 1.

Chapters III, XII, XIII, and XVI have similar, high medians compared to preceding chapters but show a different distribution, higher IQR, and long lower whiskers. Chapter XVI is the tallest box plot compared to all other chapters, with an IQR covering the entire range of confidence. Chapter XV has the lowest median, with a value around 0.3 of confidence and long upper and lower whiskers, indicating that the model’s predictions on this chapter are more variable. Except for Chapter VIII, which is used to select UCOD, and Chapters XV and XVI, whose whiskers cover the entire range, all other chapters have predictions over the entire range of confidence as outliers.

**Figure 4.**
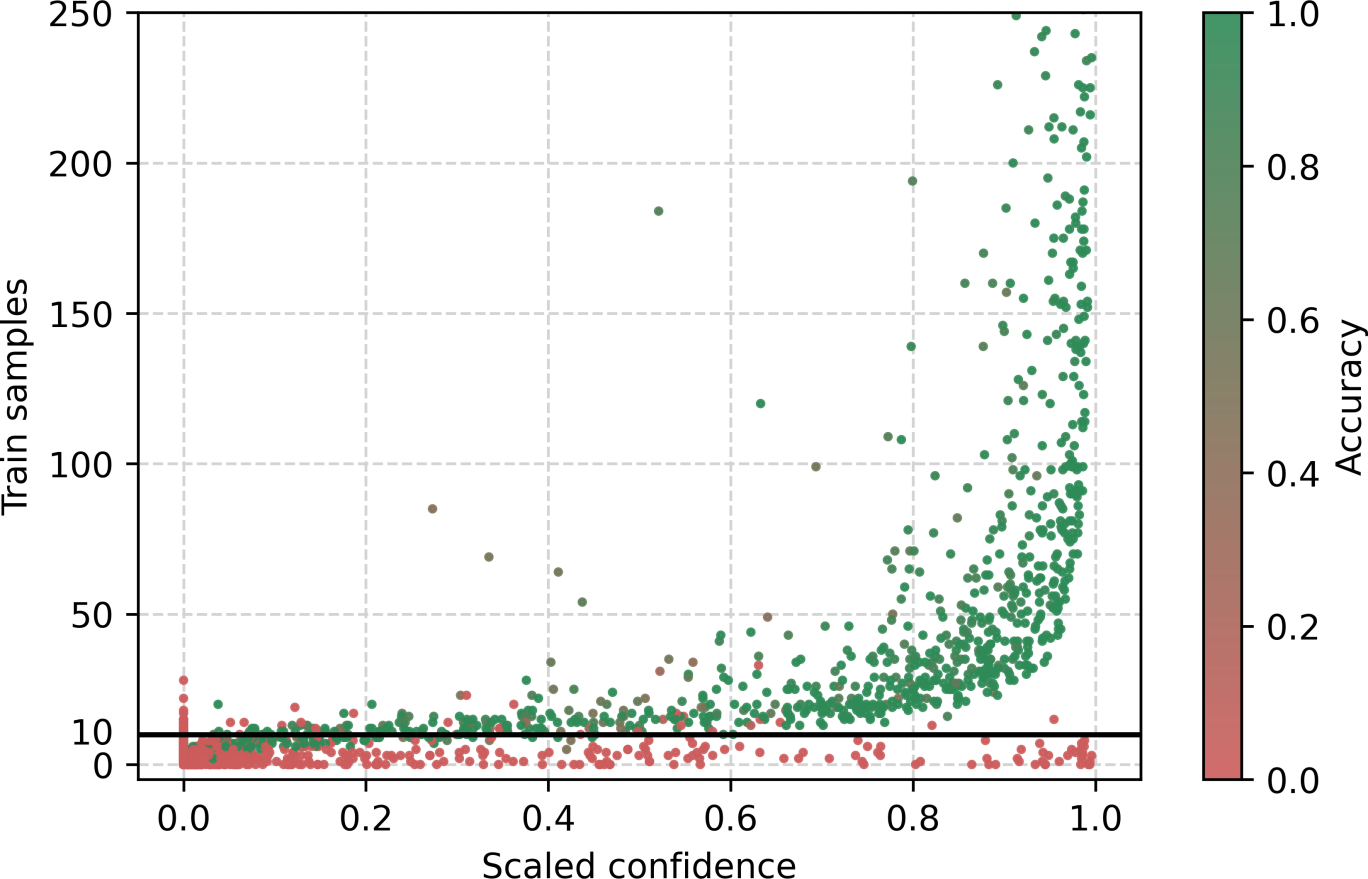
Class-wise confidence with training sample.

Figure 4 shows the distribution of training sample classes in relation to scaled confidence, with color representing class accuracy from red (accuracy of 0) to green (accuracy of 1). This variation of color will be used for multiple figures to show the accuracy. Two regions can be identified, divided by a linear function of the training samples with a value around 10.

### Instance Difficulty Estimation

**Figure 5.**
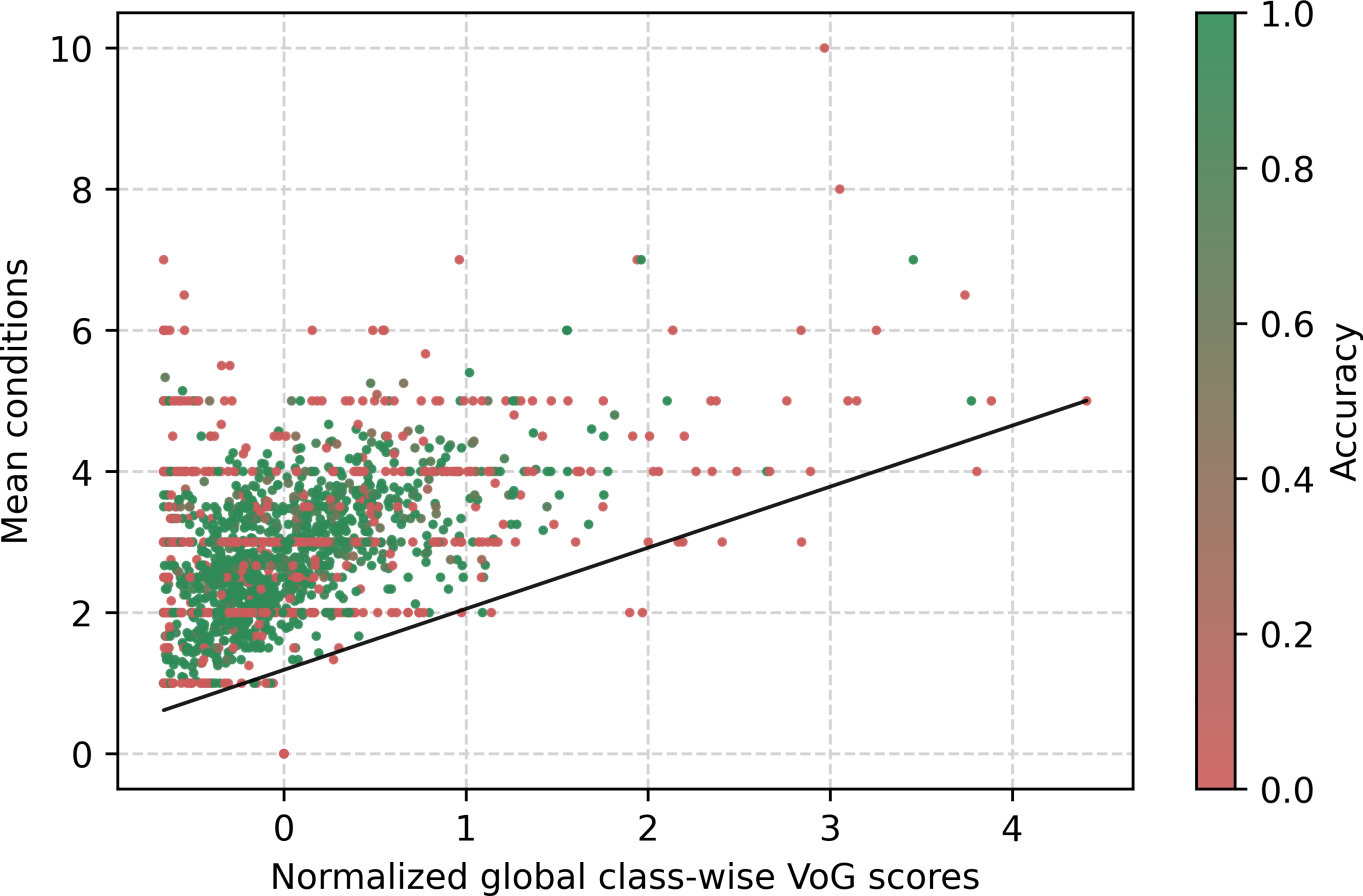
VoG globally normalized per class-wise in relation to the average condition stated in the death certificate per class. VoG: Variance of Gradients.

**Figure 6.**
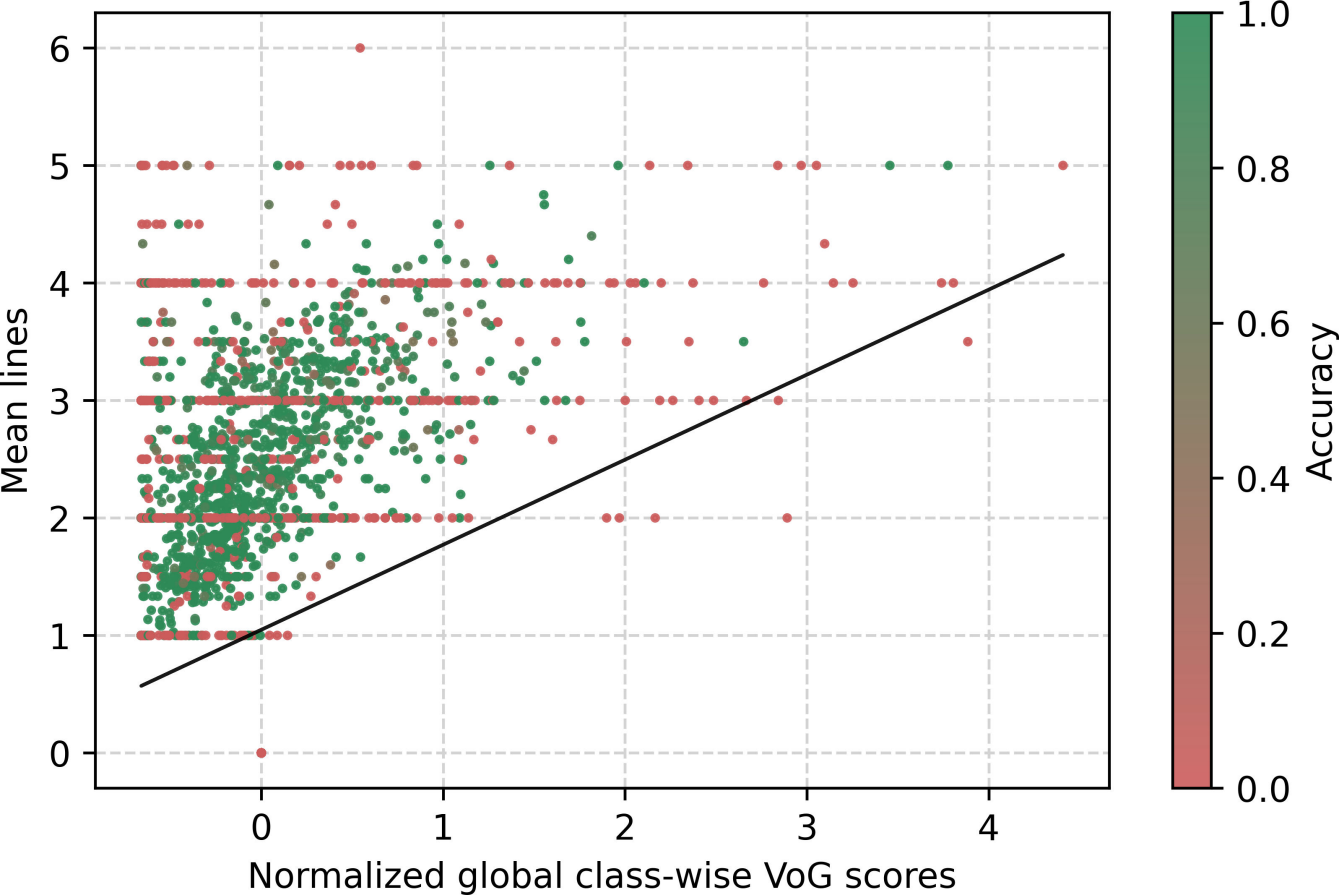
VoG globally normalized per class-wise in relation to the average lines filled in the death certificate per class. VoG: Variance of Gradients.

Figure 5 displays the mean number of conditions per class in relation to the global normalized VoG score. Each certificate may contain from 1 to 15 conditions. VoG is standardized using the global mean and SD and then clipped to the [−1,1] range for visualization; values closer to ±1 indicate higher gradient variability. The figure shows a correlation between the number of stated conditions and the difficulty of estimating the instance. When only a single condition is present, we observe that almost every instance score is below the average. On the other side, as the number of reported conditions increases, there is a corresponding increase in the average score. This indicates that instances with a greater number of conditions are generally associated with higher difficulty levels. A similar trend can be seen in Figure 6, which shows the average number of filled lines in the death certificate per class in relation to the instance difficulty VoG score for the same class, since data are most likely present in the top-left quadrant. As the average lines used per class increase, so does the difficulty. The lines displayed in the figure are simple regression lines. As an additional analysis, we computed and compared the Pearson ρ and Kendall τ correlation coefficients between VoG and the specified conditions. The correlations reveal a weak positive linear correlation between the VoG scores and both the mean conditions and mean lines, which show, respectively, Pearson ρ scores of 0.23 and 0.22 and a Kendall τ correlation coefficient of 0.06 and 0.05.

**Figure 7.**
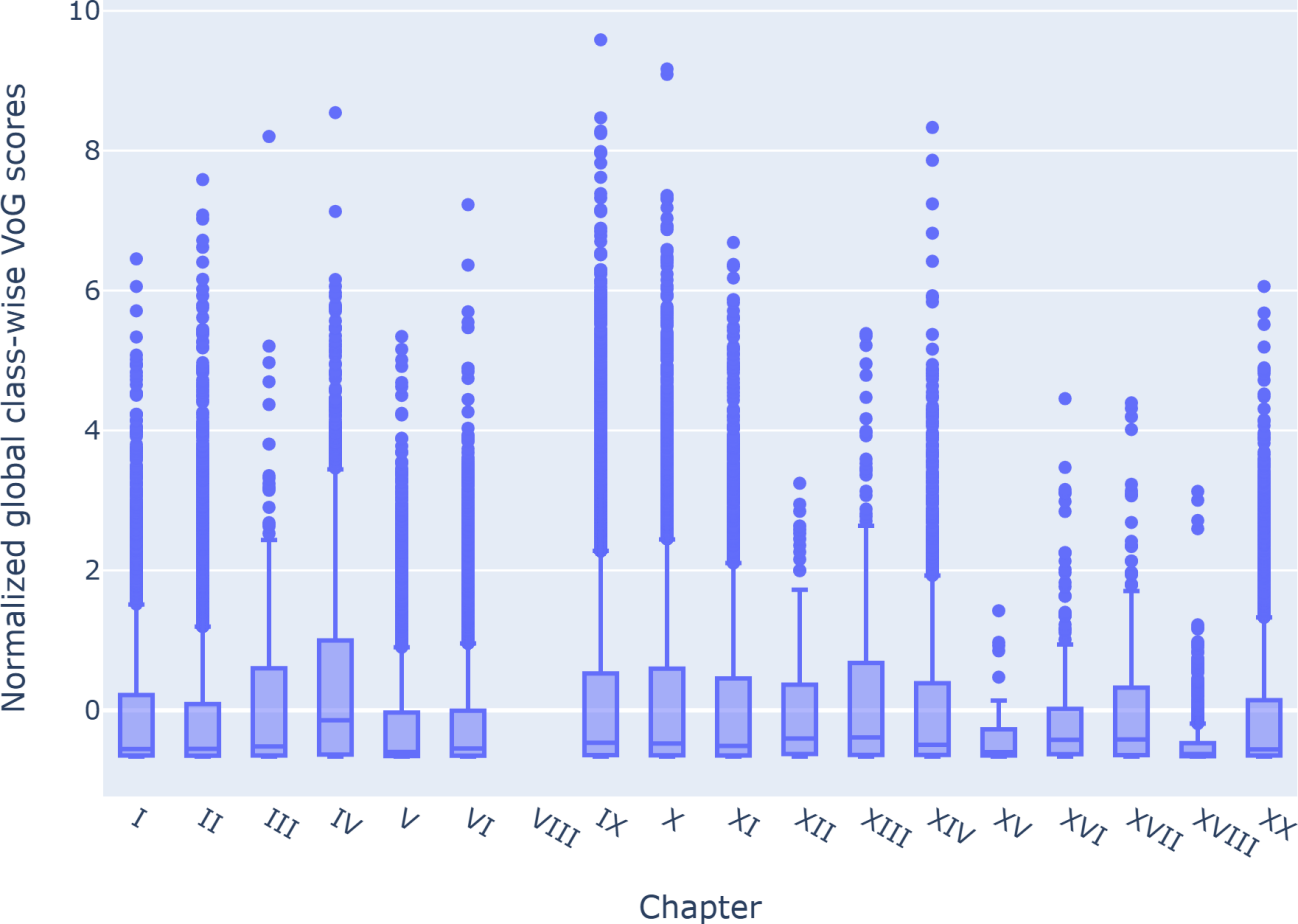
VoG globally normalized percentiles per chapter. VoG: Variance of Gradients.

The global normalized distribution of the VoG score of instances by chapter is visually displayed in Figure 7. This figure shows the data quartiles and averages, indicating that the difficulty of instances is mostly low for all chapters. Notably, the median for each chapter is lower than its corresponding average for the VoG difficulty score. Additionally, the lower quartile for all chapters is proximal to the lower value score. These observations suggest that estimating the difficulty of instances is challenging for all chapters. Among the chapters, Chapter XVIII has the lowest difficulty scores, as indicated by its upper quartile, which has the lowest value among all chapters. Furthermore, Chapters V, VI, XV, and XVI have an upper quartile lower than the average difficulty score, indicating that the instances in these chapters are less difficult.

**Figure 8.**
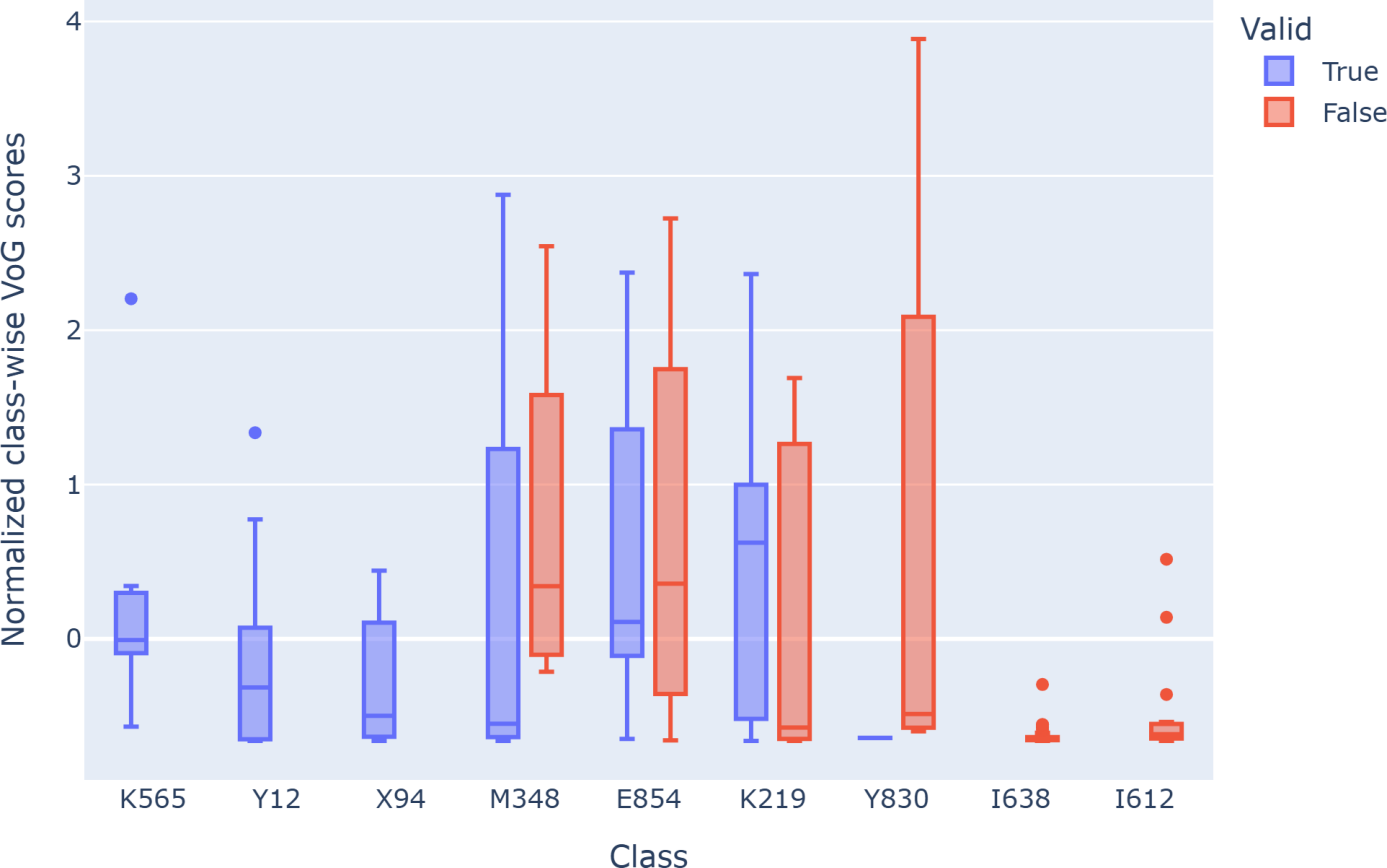
VoG globally normalized percentiles for some classes. VoG: Variance of Gradients.

We further investigated the difficulty of the instances at the class level by selecting 9 classes with at least 10 instances in the test set, sorted by error rate. Figure 8 shows the global normalized class-wise VoG score distribution for these classes. We divided these classes into 3 groups: positive classes (K565, Y12, and X94) with mostly positive predictions, mixed classes (M348, E854, and K219) with balanced predictions between positive and negative, and negative classes (Y830, I638, and I612) with mostly negative predictions. Notably, positive classes have an upper quartile lower than the mixed classes, indicating that classes with high accuracy may have fewer difficult instances compared to mixed classes. However, estimating the difficulty of classes with mostly negative predictions is challenging due to the shadowing effect of other classes. For instance, the instances of classes I638 and I612 have very low difficulty scores, contrary to our expectations based on the behavior of class Y830.

**Figure 9.**
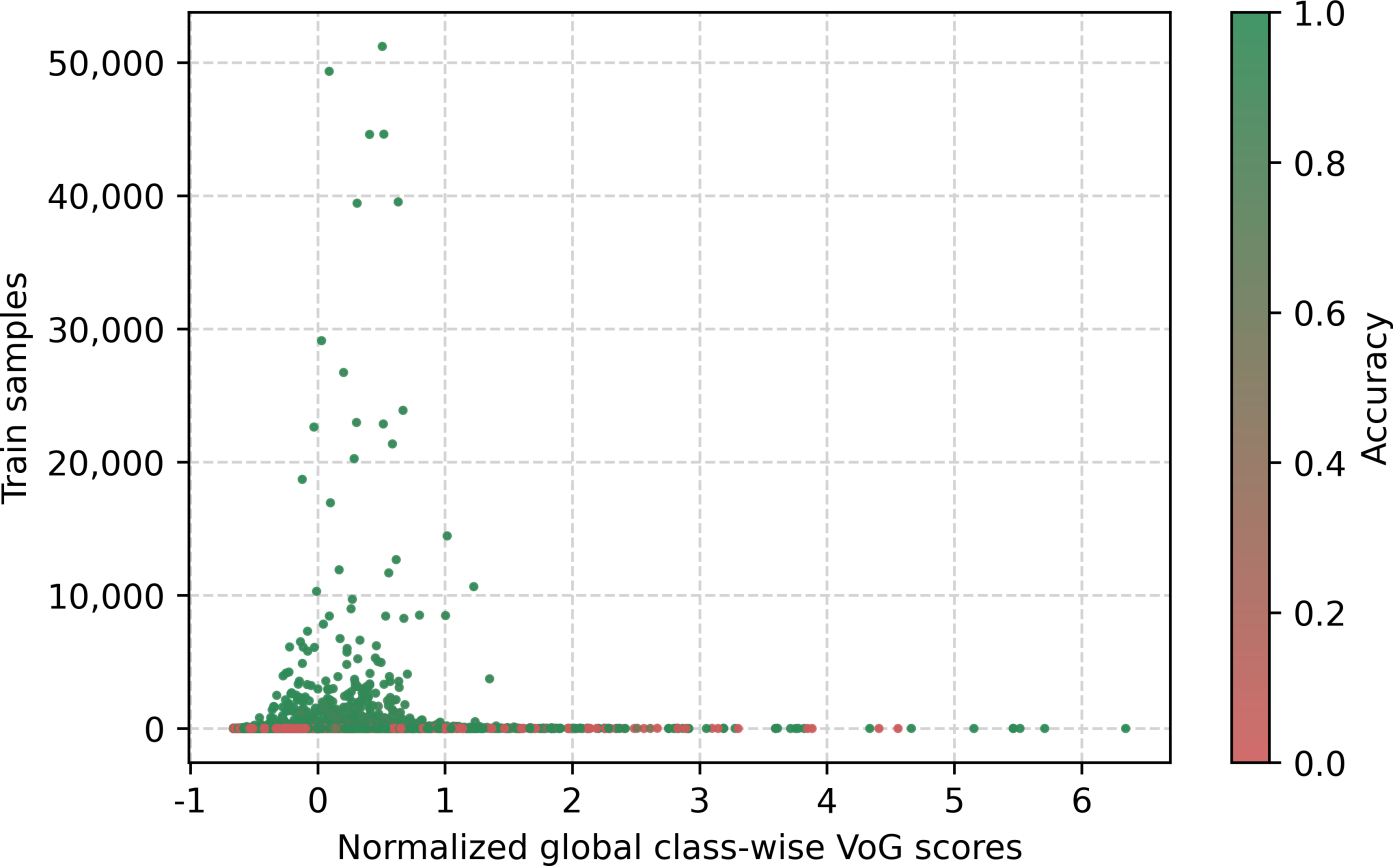
VoG globally normalized per stated condition in relation to the training sample. VoG: Variance of Gradients.

**Figure 10.**
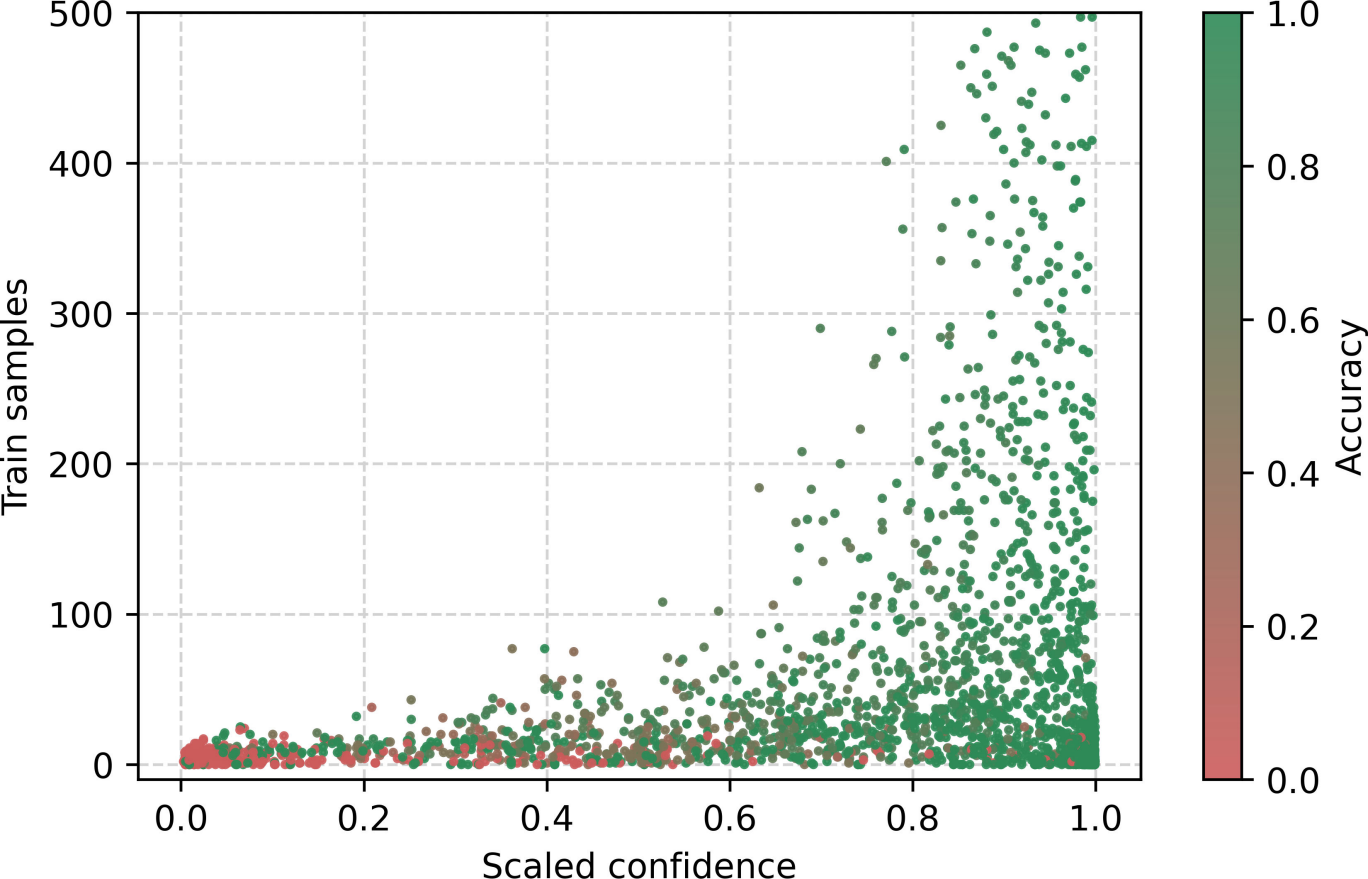
Conditions in the training sample in relation to the scaled confidence.

In addition to studying the model’s behavior with respect to the predicted class, we also explored its behavior based on the conditions stated on each certificate. Figure 9 displays the distribution of the stated conditions as a function of the globally normalized average VoG score of death certificates containing a given stated condition. A long tail pattern is observed, where less frequently stated conditions are associated with higher average instance difficulty, while conditions with a larger number of instances show more stable difficulty estimates. Figure 10, similar to Figure 4, shows the distribution of the stated conditions as a function of the scaled confidence. However, the observed behavior differs from that in Figure 4, which displays the predicted class in relation to the scaled confidence. Notably, when considering predicted classes in the long tail of the distribution, these classes have an average accuracy of 0. In contrast, low-frequency stated conditions do not necessarily influence the prediction outcome, as some conditions may not be involved in the final UCOD decision. In fact, we observed several low-frequency conditions with high confidence and low frequency that had high accuracy. However, for rare conditions with low frequency, a clear distinction between the classes that are unlikely to be predicted correctly and those that are well predicted is not evident. The results suggest that the performance of the model varies based on the conditions stated on the certificate and their frequency and role in the decision process.

**Figure 11.**
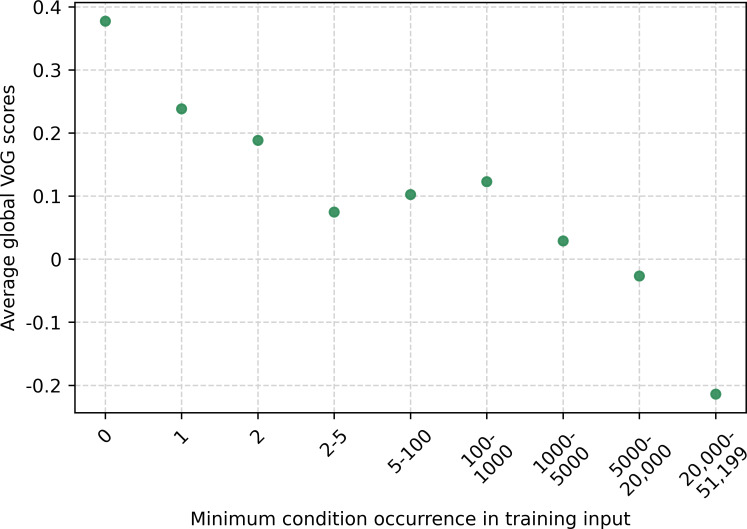
The condition with minimum occurrence in training with the average VoG score for the range. VoG: Variance of Gradients.

Figure 11 shows how the global normalized VoG score varies with the minimum occurrence of each stated condition in the training data using discrete frequency ranges. Conditions with 0 occurrences in the training set exhibit the highest difficulty, with an average VoG score of approximately 0.4. When the minimum occurrence increases to 1 or 2 instances, the average VoG score remains elevated (around 0.2). For conditions appearing between 2 and 1000 times, the VoG score decreases but remains positive (≈0.1). Conditions in the higher frequency ranges (1000‐5000 and 5000‐20,000 occurrences) show VoG scores close to 0. A notable change is observed for conditions occurring more than 20,000 times, where the average VoG score becomes negative (≈–0.2), suggesting that these instances are highly redundant and therefore easier. Overall, the results indicate that instance difficulty decreases as condition frequency increases.

**Figure 12.**
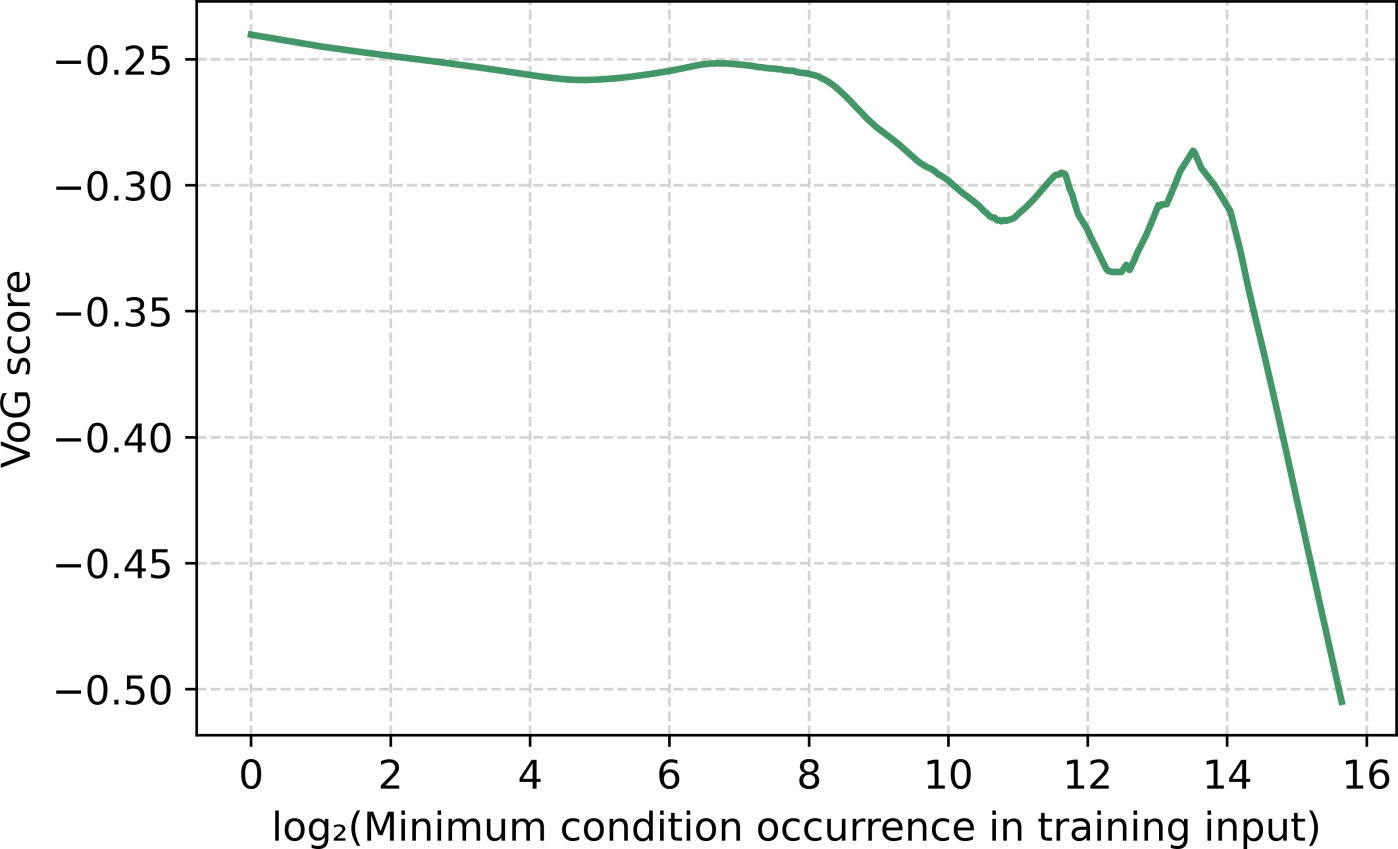
Minimum condition occurrence in the training data (log_2_-scaled) versus the corresponding VoG score. A LOESS regression line is used to smooth the trend across the full range of frequencies. LOESS: locally estimated scatterplot smoothing; VoG: Variance of Gradients.

Figure 12 presents the same relationship as Figure 11 using a log_₂_-transformed axis with locally estimated scatterplot smoothing. The trend shows a gradual decline in difficulty as condition frequency increases, with VoG values decreasing slightly up to log_₂_≈12. Between log_2_≈12 and 14, the curve stabilizes. Beyond log_2_≈14, the difficulty decreases more sharply, with estimated VoG values dropping from approximately –0.30 to –0.50 by log_2_≈16. This pattern indicates a pronounced reduction in difficulty for conditions with very high training frequencies.

### Saliency Maps Overview

**Figure 13.**
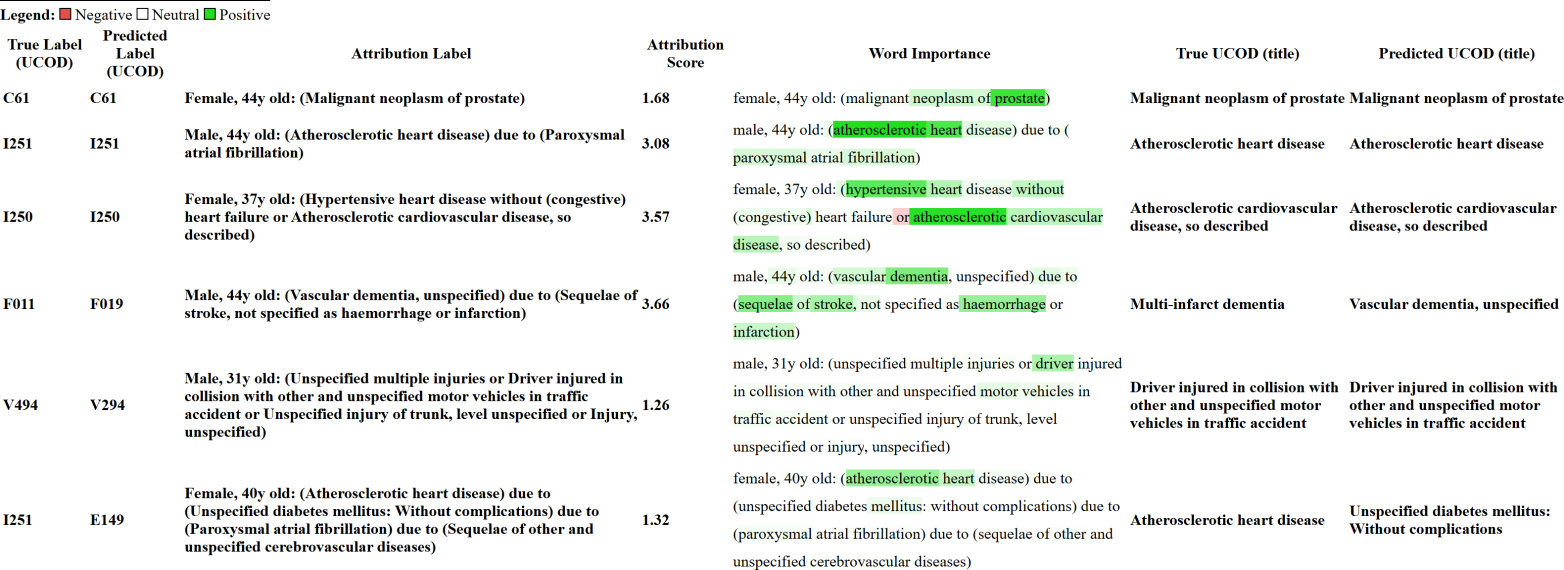
Word importance with attribution scores computed using Layer Integrated Gradients for 3 positive cases and 3 failure cases, as analyzed using Captum. UCOD: underlying cause of death.

In Figure 13, we present an overview of saliency maps computed using Captum. The figure includes 3 positive examples (the first 3 rows) and 3 failure cases (the last 3). For each case, the true and predicted UCOD codes are shown in the “True Label (UCOD)” and “Predicted Label (UCOD)” columns, respectively, while the corresponding input text is provided in the “Attribution Label” column. The “Word Importance” column displays the attribution assigned to each word, representing the key results from the saliency maps. In this visualization, red indicates a negative attribution score, white indicates a neutral score, and green indicates a positive score, with positive values highlighting words that contribute more strongly to the classification decision. The “Attribution Score” column reports the sum of all word-level attributions, which is generally greater than 0, although in some cases, the total may be negative. Additionally, the titles corresponding to the true and predicted labels are shown in the “True UCOD (title)” and “Predicted UCOD (title)” columns.

## Discussion

### Principal Findings

In the medical domain, as in other mission-critical areas, the black-box nature of ML models limits their adoption in real-world applications. This is because users need to understand what the model is doing, why a specific result is produced, and when their intervention may be necessary due to unreliable outcomes. In the specific use case presented here, the starting point was a model that provides better accuracy compared with the standard approach, yet is considered not usable in practice.

In this study, we proposed 3 approaches to analyze and improve the reliability of the model. First, we evaluated the model’s calibration using ECE and MCE, conducting this analysis at both the chapter level and the category level by considering the distribution of training samples and the frequency of inputs and labels. Next, we presented the VoG as a ranking framework to identify the most challenging instances and to support OOD analysis in this work. In this context, OOD does not refer to explicit distributional shifts between training and test data, nor to unseen UCOD labels, as the train-test split was performed using stratified sampling over the target classes. Instead, OOD is used in a task-specific sense to describe rare labels and rare or previously unseen ICD codes stated on the certificate. While certain code combinations and positional effects may influence the rule-based UCOD selection process, such cases cannot be explicitly identified in our analysis. We carried out the same chapter-level and category-level analysis for the VoG scores to better characterize such behavior. This analysis helps experts gain greater control over the model’s behavior. Finally, we introduced saliency maps to provide word-level attribution and highlight the importance of specific input features.

The complexity of the proposed methodologies is relatively low. The training of the model proposed by Della Mea et al [26] requires approximately 8 hours, while inference takes only a few minutes. Additionally, the integration of temperature scaling and the computation of the VoG can be easily implemented on the trained model. Specifically, the temperature scaling technique proposed by Guo et al [1] involves dividing the logits (inputs to the softmax function) by a learned scalar parameter, simplifying the calibration process. Meanwhile, the computation of the VoG score, proposed by Agarwal et al [15], is straightforward and quick to perform on the test set. This is because it relies solely on the vanilla gradient explanation from the model, making the process efficient and accessible, and the same goes for the saliency maps computation.

Although transformer models have been shown to be well calibrated [34], we demonstrated that our model is calibrated out of the box with respect to the ECE but less so for the MCE, which reflects the worst-case deviation across bins (Figure 1). When applying temperature scaling, a standard post-hoc calibration method, we observed a small improvement in ECE but a considerable increase in MCE. A closer inspection indicates that this trade-off is largely driven by the extreme imbalance in the confidence distribution. Because the model achieves an accuracy of 0.990, almost all predictions fall within the highest confidence bin between 0.9 and 1.0, while the remaining bins contain only a very small number of samples. Consequently, temperature scaling improves the average calibration but amplifies errors in sparsely populated bins, which disproportionately affects the MCE. This suggests that temperature scaling may not be suitable for improving calibration when confidence bins are strongly unevenly distributed, and that the increased worst-case error should be interpreted with caution. In our specific use case, we place greater importance on maintaining good calibration in the 0.9‐1.0 confidence range, where the vast majority of predictions lie, and we treat the remaining lower-confidence cases as candidates for manual review. Under these conditions, the improvement in ECE obtained through recalibration can still be considered beneficial.

From the analysis conducted at the chapter level (Figure 3), we found that some chapters, such as XV (Pregnancy, childbirth, and the puerperium) and XVII (Congenital malformations, deformations, and chromosomal abnormalities), have overall low confidence. This is expected, as these conditions occur very infrequently in the dataset (Table 3). In practice, existing rule-based systems also reject these cases, and all certificates would be subject to manual review because they are highly sensitive. Additional attention may also be needed for Chapters III (Diseases of the blood and blood-forming organs and certain disorders involving the immune mechanism), XIII (Diseases of the musculoskeletal system and connective tissue), and XVI (Certain conditions originating in the perinatal period), which exhibit a large IQR together with a low first quartile. These patterns likewise reflect the low target frequency observed for these chapters (Table 3).

Figure 2 also presents the relationship between confidence and error rate at the chapter level. In the top-right corner, we observe a cluster of chapters with an error rate of 1, reflecting the fact that these chapters are never predicted; in particular, Chapter XIX (Injury, poisoning, and certain other consequences of external causes) cannot be selected as the UCOD. In contrast, the lower-right region shows a grouping of chapters with both high accuracy and high confidence. The remaining chapters fall outside these clusters and would be candidates for manual intervention or for targeted improvements in the training data. Additionally, we manually investigated specific training samples in relation to confidence (Figure 4) and found that classes with fewer than 15 training samples have very low accuracy, which can be visually identified in the bottom region. From the analysis at the chapter level and based on the distribution of training samples, we can clearly distinguish instances that require further improvements and, when this is not feasible, identify cases that should be flagged for manual selection by experts.

Figures5  6 present the VoG score in relation to the average number of lines and the average number of conditions stated on the certificate. Although we can visually observe an increase in instance difficulty as the number of conditions and lines increases, the correlation between these features appears to be weak. Figure 7 shows the VoG distribution at the chapter level, and Figure 8 illustrates the VoG for a selection of individual categories. Overall, the average VoG remains low across chapters, but we observe a substantial dispersion with several high-VoG outliers. These cases, observed at both the chapter and category levels, should be taken into consideration for further investigation and may indicate instances that require additional training data or manual review.

Furthermore, we extended our investigation to the combined analysis of VoG and confidence with respect to the number of conditions stated as input in the death certificate, as shown in Figures 9-12. In particular, we observe that failure cases are distributed differently when examined in relation to the input conditions compared with the distribution based on the label frequency in the training set. This behavior is expected, since the conditions listed on the certificate are not always necessary or directly relevant for determining the UCOD. In Figure 11, we note that the average VoG is considerably higher when the input includes conditions that were either absent or occurred only a few times in the training data. Additionally, the difficulty decreases more sharply after a log_2_ value of approximately 14 (Figure 12).

In particular, Figure 9 shows that VoG is able, to some extent, to highlight OOD-like behavior, as reflected by a long tail of high-difficulty classes corresponding to conditions that appear only a few times in the training set. More clearly, the average VoG is highest for cases involving codes that never appear in the training data (Figures11  12).

In Figure 13, we present an overview of how saliency maps can support model interpretability. Specifically, saliency maps highlight which parts of the input text are most influential for the model’s decision-making process by identifying words or conditions that contribute most to the predicted UCOD. In the 3 positive examples, selected to illustrate common WHO-rule scenarios, we observe a close correspondence between the predicted label and the features receiving the highest attribution. In the first example, the certificate contains a single condition, a frequent real-world scenario. The attribution correctly emphasizes “prostate” more strongly than “malignant neoplasm,” reflecting the fact that many malignant neoplasm categories share similar wording and rely on the anatomical location for discrimination. In the second example, 2 conditions are reported on separate lines, and the first is selected as the UCOD. This is expected because atrial fibrillation and flutter (I48), when mentioned alongside “ischemic heart disease” (I20-I25), must be coded within the “ischemic heart disease” range. Accordingly, the saliency map assigns meaningful attribution not only to the first condition but also to the supporting condition needed to justify its selection. In the third example, the second condition on the first used line is selected as the UCOD, but this selection is only valid in the presence of the first condition on the same line. The saliency map reflects this rule-consistent structure by highlighting both conditions. Across these examples, we can discern which parts of the input are most decisive for the classification and how the model appears to rely on patterns aligned with underlying WHO rules.

The final 3 examples in Figure 13 illustrate failure cases. In the first case, the model predicts “vascular dementia, unspecified” as the UCOD. Although this condition is less specific than the correct one, it corresponds to the condition explicitly stated on the certificate. The correct UCOD is obtained only when this condition is recoded into a more specific category based on the combination of conditions mentioned. However, such recoding patterns are absent or extremely rare in the training data, preventing the model from learning this specific transformation. As a result, the attribution pattern is logically consistent with the input representation, even though the final prediction is incorrect. In the second failure case, the error arises because 2 distinct ICD-10 categories share the same title, an issue present in only a few parts of the classification. The reverse-coding process used for training collapses these distinct codes into an identical textual representation, thereby removing the information required for the model to distinguish between them. As a consequence, the model cannot learn this distinction, regardless of reasoning quality. The saliency map nonetheless assigns meaningful attribution to terms such as “driver” and “motor vehicle,” which is consistent with the underlying coding logic: injuries cannot be selected as UCOD, and external causes take precedence when present. Since the injury itself is not part of the causal sequence leading to the UCOD, it is plausible that it receives little or no positive attribution. In the third case, the model assigns a positive attribution to the condition that corresponds to the true UCOD, while giving only a slightly positive attribution to the condition it incorrectly predicts. Here, the attribution appears incongruent with the predicted label, and we would have expected to see clearer importance assigned to both the last condition and the predicted UCOD. This discrepancy highlights a mismatch between attribution and prediction, which aligns with the fact that the model selects an incorrect UCOD.

### Limitations

In our analysis, we presented statistics on the ICD-10 codes actually present in the training dataset, which encompass approximately 36% of the entire ICD-10 classification. This suggests that one of the primary limitations of the trained model is the coverage of the codes in the dataset, potentially contributing to errors in our predictions on the test set. Consequently, understanding OOD data is crucial, as we attempted with our analysis.

Additionally, we consider that errors may occur due to incomplete input data. As described by Della Mea et al [26], while the certificate input is textual, the dataset contains coded data rather than plain textual descriptions. Thus, the data used for training are derived from a reverse coding process from the code to the title of the condition. Unlike a rule-based system where coders can access the full certificate, our study evaluated only the administrative data and conditions stated in the certificate, as this was the only data available. Consequently, there are instances where the same certificate with the same input conditions may assign a UCOD with different codes, which our model cannot differentiate without access to the comprehensive death certificate knowledge used by the coders.

Our calibration analysis is based on a single post-hoc method, temperature scaling, and reveals a trade-off between average and worst-case calibration. While the model is reasonably well calibrated out-of-the-box in terms of ECE, the MCE remains higher, and applying temperature scaling further improves ECE at the cost of worsening MCE. Given the very high accuracy of the model and the strong concentration of predictions in the highest confidence bin, this behavior suggests that temperature scaling may not be ideal in settings with such imbalanced confidence distributions.

There are also limitations in how VoG and saliency maps capture instance difficulty and explainability. VoG shows only a weak correlation with simple difficulty features such as the number of conditions or lines, meaning it does not fully reflect these intuitive measures, even if it remains helpful for identifying rare or OOD instances. Saliency maps were illustrated through a small set of positive and negative examples, and, given that the input consists of reverse-coded representations based on standardized ICD concept titles rather than natural clinical narratives, token-level attributions reflect the informativeness of specific standardized terms rather than linguistic nuance in unstructured text. Token-level attributions cannot provide a full explanation for a task governed by complex WHO rules. They offer useful insights into which parts of the input influence the model’s decisions but should be viewed as complementary rather than exhaustive explainability tools.

### Future Work

Future research should evaluate the model on data that differ substantially from the training distribution, such as certificates from the COVID-19 period, to assess how well confidence and VoG scores identify emerging or atypical cases. A more detailed investigation of very high-VoG instances, conducted together with domain experts, is also needed to understand why these cases are difficult and whether they can guide improved training strategies, for example, through difficulty-aware sampling or adaptive instance weighting.

Further work is required to deepen the analysis of saliency maps and to determine, through expert feedback, whether token-level attributions provide explanations that are meaningful and actionable in practice. More generally, open questions remain regarding how structural elements of the input representation influence gradient-based explanations and whether such effects reflect model internals, methodological artifacts, or data-dependent interactions. Addressing these issues will be important to improve the robustness and interpretability of attribution-based explanations. Finally, alternative calibration approaches that maintain high ECE performance without increasing MCE should be explored.

### Conclusions

This study evaluated the interpretability of a transformer-based model for UCOD identification by jointly analyzing confidence calibration, instance difficulty, and input-level explanations. Using a pre–fine-tuned model on large-scale reverse-coded death certificate data, we showed that the model exhibits good out-of-the-box calibration in terms of ECE, while MCE remains higher. Post-hoc temperature scaling further improves average calibration but increases worst-case deviations, indicating that calibration methods should be applied cautiously in highly imbalanced confidence regimes.

VoG provides a complementary signal for ranking instance difficulty, supporting the identification of potentially OOD cases that may require expert review. Although VoG shows limited correlation with simple difficulty proxies, it contributes additional information beyond confidence scores alone. Saliency maps based on gradient attribution highlight input tokens influencing model predictions and reveal patterns consistent with established WHO coding rules in both correct and incorrect cases. Overall, these combined analyses create a structured framework for interpreting transformer-based clinical coding models.

## Abbreviations

BERT: bidirectional encoder representations from transformers
DiLBERT: disease language bidirectional encoder representations from transformers
ECE: expected calibration error
ICD: International Statistical Classification of Diseases and Related Health Problems
ICD-10: International Statistical Classification of Diseases and Related Health Problems version-10th revision
ICD-11: International Statistical Classification of Diseases and Related Health Problems- 11th revision
ID: in-distribution
IG: Integrated Gradients
MCE: maximum calibration error
ML: machine learning
NN: neural network
OOD: out-of-distribution
UCOD: underlying cause of death
VoG: Variance of Gradients
WHO: World Health Organization
